# SSRI'S and other antidepressant use during pregnancy and potential neonatal adverse effects: Impact of a public health advisory and subsequent reports in the news media

**DOI:** 10.1186/1471-2393-5-11

**Published:** 2005-05-20

**Authors:** A Einarson, AK Schachtschneider, R Halil, E Bollano, G Koren

**Affiliations:** 1 The Motherisk Program, The Hospital for Sick Children, Toronto, Canada; 2 Department of Pharmacoepidemiology & Pharmacotherapy, Utrecht Institute for Pharmaceutical Sciences, Utrecht, The Netherlands; 3 The Leslie Dan Faculty of Pharmacy, University of Toronto, Toronto, Canada

## Abstract

**Background:**

On Aug 9^th ^2004 Health Canada released an advisory, which followed a similar one from the FDA regarding the use of SSRI's and other antidepressants during pregnancy and potential adverse effects on newborns. In neither advisory was it stated that women should discontinue their antidepressant. In the seven days following the release of this advisory, The Motherisk Program received 49 calls from anxious women in response to the media reporting of this information.

**Objective:**

To examine the impact of the advisory and subsequent reporting in the media, on the decision-making of women, currently taking an antidepressant, who called The Motherisk Program after becoming aware of this information.

**Methods:**

We attempted to follow up all the women who had called us who were alarmed by this advisory and asked them to complete a specially designed questionnaire.

**Results:**

We were able to complete 43/49 (88%) follow-ups of the women who contacted us. All of the callers reported that the messages in the media caused a great deal of anxiety. Seven misunderstood the advisory, ie their children were more than 1 year old, five had discontinued their antidepressant (3 abruptly (2 later restarted after speaking with Motherisk counsellors)and 2 with some form of tapering off) and(6) were considering discontinuation, but decided to continue following reassurance from Motherisk

**Conclusion:**

Medical information regarding fetal and infant safety, disseminated in the public domain, should be transferred in a way that does not influence a pregnant woman to make decisions that may not be in the best interest of hers or her child's health.

## Background

The media plays a significant role in the dissemination of medical information to the general public, through newspapers, television, magazines and more recently through the internet. While it is important to get these messages to the public, at times it appears that the media may have a different agenda, such as selling newspapers or widening their TV audiences. They seem to prefer alarmist headlines and display a preference for stories that catch attention, rather than to inform the public of new scientific data that has both positive and negative results. For example, stories describing safe products are often unreported, or reported in small print, whereas unsafe products usually make headlines. [1] Since the thalidomide scare in the sixties, this has been especially true of stories concerning the safety/risk of exposures during pregnancy.

A recent example in another field, can be found in the study of the use of hormone replacement therapy (HRT) following the release of The Women's Health Initiative (WHI) which was widely publicized in the lay press. [2] A recent survey, completed by more than 1000 women, that examined their opinions and understanding from the lay press of this study, found that women dramatically overestimated the risks of HRT.[3] Another study found that information on the benefits of HRT was mainly provided by health care professionals, whereas information on the risks was provided mainly by the mass media. [4]

The Motherisk Program, at the Hospital for Sick Children in Toronto, is a counseling service that provides pregnant, breastfeeding women, and health care providers with evidence-based information on the safety and risks of exposures to prescription and over-the-counter (OTC) medications, natural health products, chemicals, radiation, and infectious agents. Women and their health care professionals often call us when alarming stories regarding pregnancy exposures are reported in the media. Consequently, we receive a dramatic increase in the number of calls to our service following a report in the media that involves a study that has produced results that are distressing to a pregnant woman.

On June 9^th ^2004 the FDA(Food and Drug Administration) instructed the manufacturers of antidepressants to issue warnings about perinatal complications associated with their products. FDA: Medwatch Drug Alert. June 03/2004). On Aug 9^th ^2004, Health Canada, followed suit and released on their website  an advisory warning of the potential adverse effects of Selective Serotonin Reuptake Inhibitors (SSRI's) and other antidepressants on newborns. It was stated that the advisory was intended to increase awareness among mothers and physicians of the possible symptoms that may occur in the newborn, so that they can be recognized and addressed quickly. Some of the symptoms listed were from reports describing feeding and/or breathing difficulties, seizures, muscle rigidity, jitteriness and constant crying. The advisory also stated that if a woman is pregnant and taking an antidepressant she should discuss with her health care professional the potential benefits and risks of treatment options. It was also stated that it is very important that women do NOT stop these medications without consulting their doctor, however, physicians may consider slowly decreasing the dose in the third trimester of pregnancy. It must be noted, that nowhere in this advisory was it stated that women should avoid taking antidepressants during pregnancy. [5]

This advisory was reported in all forms of the media. Table 1 shows some examples, which were selected both randomly and from the women's reports.

**Table 1:** Samples of media reporting of advisory (headlines)

Radio: (Ottawa) "Antidepressants pose risk to unborn babies"
Internet: (Canada.com News) "Depression drugs can hurt babies"
Newspaper: (The Toronto Star)"Avoid antidepressants in pregnancy"
Television: (CTV) Antidepressants may put unborn babies at risk
Magazine: (Health and Wellness) "Pregnant women warned about antidepressants"

In the seven days following the media reports, we received 49 calls to the Motherisk line from anxious women. This number was in addition to the already substantial number of calls regarding the use of antidepressants we receive each day. We were not surprised that there was this sudden influx of calls, as we felt that this advisory was clearly ambiguous and it was understandable that the media may have misinterpreted some of the intended message.

The objective of this study was to examine the impact of the media translation of this advisory, on the decision-making of women who were currently taking an antidepressant and called Motherisk for information.

## Methods

Researchers at the Motherisk Program developed a questionnaire that consisted of 4 questions, (Appendix). Potential participants were women currently treated with an antidepressant who had contacted the Motherisk Program after hearing about the Health Canada advisory in the media and becoming alarmed.

After receiving evidence based information provided by a Motherisk counselor, (table 2) each woman was followed up by phone within one week of their initial call. Upon contact, she was asked if she would be willing to participate in a telephone survey to be conducted by a student. Oral consent was obtained from each participant after the survey was fully explained over the telephone. If the interviewer was unable to reach the women on the first contact, two more attempts were made before giving up.

**Table 2:** Evidence-based information given by Motherisk counsellors

1. Antidepressants are an important pharmacological tool in the treatment of depression, including during pregnancy.
2. Based on epidemiologic studies they are considered an exposure that would not harm the fetus.
3. Untreated maternal depression is associated with adverse effects on both the mother and the fetus.
4. In a minority of infants there is a mostly self limited but if required, treatable discontinuation syndrome. Consequently, the baby should be observed carefully after birth for signs of withdrawal.
5. If a woman has discussed the benefits and risks of taking an antidepressant during pregnancy with her physician and if the decision is to be pharmacologic treatment, based on current epidemiologic data, there is no reason to discontinue or decrease the drug anytime during pregnancy.

The data were analyzed by descriptive statistics.

## Results

A total of 49 women agreed to participate and 43 completed the survey, a response rate of 88%. Of the women who agreed to participate but were lost to follow-up; 2 had incorrect telephone numbers (either because they had given the wrong number or it had been noted incorrectly) and 4 of the women were unavailable to complete the questionnaire. All of the callers reported that the information they received from the media caused a great deal of anxiety. They all felt that this was important information to know, however would not have been so alarmed if it had been translated by the media in a less "scary" fashion.

The following results underscore how the information was translated in such a misleading fashion. This information was specifically aimed at women taking an antidepressant in late pregnancy. Thirty-two of the women were not in the third trimester at the time they called the Motherisk Program and eleven were not even pregnant. Four of them were planning a pregnancy and seven had taken an antidepressant when they were pregnant and their children were now more than one year old. Three of the women who stopped taking their antidepressant, discontinued it abruptly, although two restarted following reassurance from Motherisk. Six women were considering discontinuing their medication, but were reassured by Motherisk and decided to continue. Table 2

## Discussion

To our knowledge, this is the first survey of it's kind that has been conducted to examine the impact of public health advisories and the subsequent reports in the media on the decision-making of pregnant women regarding taking antidepressants during pregnancy. We were able to interview a convenience sample of 43 women who had called our line following the dissemination of this information in the media. We did not set out to conduct a randomized controlled study, we simply wanted to document in an observational fashion, how the women who called our program felt about the information they had received. As such, we were unable to ascertain how many other women in the general population were also possibly negatively affected by rash decision-making prompted by these stories in the media. Conversely, we were also unable to examine how many women were unaffected by the media attention to this advisory, as they did not call us.

To date, based on a fairly substantial body of epidemiologic data, there is no evidence that antidepressants are not safe to take during pregnancy [6], in fact, emerging data in the literature documents evidence that not taking an antidepressant if it is warranted may be more harmful. The decision to discontinue taking an antidepressant during pregnancy can have deleterious effects on both the health of the mother and her baby as untreated depression during pregnancy carries its own risks. These include adverse maternal outcomes such as: poor compliance with prenatal care, inadequate nutrition, poor pregnancy outcomes including increased risk of preterm delivery and importantly, an increase risk of post partum depression.[7] Additionally, abrupt discontinuation of antidepressant medication during pregnancy can result in serious physical and psychological adverse effects, which may include substitution of alcohol for medication, acute onset of a major depressive episode and suicidal ideation. [8] Furthermore, studies of infants born to depressed mothers have reported that these infants exhibit "depression-like behavior, demonstrated by decreased facial expressions, inferior orientation skills, excitability and abnormal reflexes.[9] Conversely, the neonatal SSRI poor adaptation syndrome occurs in a minority of cases, is usually self-limited and to date there is no evidence to suggest that a woman should discontinue her antidepressant in late pregnancy.[10] However, the research is ongoing, so it is not possible to say definitively how often this occurs or that there are no long term adverse effects on the child whose mother was exposed to an antidepressant in late pregnancy. A woman and her physician should always discuss the evidence-based information on both the positive and negative effects of treatment with an antidepressant in pregnancy before making a decision, which will ensure the best possible outcome for the mother and child.

It is important that scientific information is disseminated to the public to empower them to take care of their health. Paternalistic models of health care delivery have been replaced by a more balanced approach, which includes both patient and provider goals Today, patients are encouraged to actively participate in the decision-making regarding management and treatment of their health and the various forms of the news media particularly the Internet are powerful tools to facilitate this process. However, the way in which this information is translated from scientific data and disseminated to the public must be carried out in a responsible and circumspect fashion. Shuchman et al. documented the four problem areas in the reporting of medical information to the public by journalists: sensationalism, biases and conflicts of interest, lack of follow-up and stories that are not covered. [11] Motherisk has previously shown that studies reporting results that showed no harmful effects, were underreported as compared to studies that reported results that showed harmful effects, for example, the use of cocaine in pregnancy.[12]

The major limitation of this study, is that we do not know how this media translation of scientific information affected the general population of pregnant women who are taking antidepressants during pregnancy for example, women who did not call Motherisk. It remains unclear how many women may have suffered from preventable adverse events secondary to abrupt discontinuation of their antidepressant as a result of these news stories, as we only have information regarding the women who did call The Motherisk Program. On the other hand some women may have been unaffected by these stories and continued to take their medication without any concerns.

In summary, this is an example of how the news media in their misguided reporting of information from a Health Canada Advisory, which in itself was rather ambiguous, influenced a number of pregnant women to make decisions that may not have been in the best interests of their health or the health of their unborn child.

## Appendix

Questionnaire

1. Were you taking an antidepressant when you called Motherisk for information?

YES NO

2. If no:

1) Did you stop taking the medicine:

Abruptly Gradually (i.e. taper off)

2) After speaking with Motherisk, did you restart the antidepressant?

YES NO

3. After speaking with Motherisk, did you speak with your MD?

1) YES NO

2) If Yes:

Did your MD recommend you continue to take the antidepressant?

YES NO

3) If No, why?__________________________________________

4) If Yes:

Did you continue to take the antidepressant?

YES No

4. In your own words, "what impact do you feel that the media stories had on your decision making regarding whether you took the antidepressant or not"?

**Table 3:** Background of the women who called Motherisk

N = 43	
Retrospective	7
Planning	4
Not in 3rd trimester	32
Discontinued drug (3 abruptly)	5
Considered discontinuing drug but did not	6
Recommendations to discontinue (MD)	5

## Pre-publication history

The pre-publication history for this paper can be accessed here:


